# Intrahospital infection as a risk in haemodialysis patients with central venous catheters

**DOI:** 10.1186/1753-6561-5-S6-P207

**Published:** 2011-06-29

**Authors:** V Gerasimovska, K Popovska-Jovanovska

**Affiliations:** 1 University Clinic of Nefrology, Medical Faculty, Skopje, Macedonia, The Former Yugoslav Republic Of; 2 Institute for Microbiology and parasitology, Institute for Microbiology and parasitology, Medical Faculty, Skopje, Macedonia, The Former Yugoslav Republic Of

## Introduction / objectives

Central venous catheters (CVC) are routinely used in the management of haemodialysis (HD) patient (pts). One of the most frequently encountered complications is catheter-related bloodstream infection.

## Methods

We describe a HD pts who developed *S. maltophilia* bacteremia associated with use of CVC.

## Results

During two years period five pts (3 male, 2 female) on chronic HD program were admitted to our hospital with clinical signs of CRBI.Three pts had tunneled subclavian catheter, one tunneled jugular catheter, and one femoral catheter. Duration time of chronic HD program was between 1 month and 5 years, and duration time of CVC was 1-18 months. All pts had clinical symptoms of high fever and chills during or after the HD, and we took blood cultures from catheter and peripheral vein and antibiotics were used. Incubation of blood cultures for 48 hours yielded bacterial growth of S. maltophilia. A complete blood count in all pts revealed a white blood cell count of 7,550-24,000 cells/mm^3^ (70-90% polymorphonuclear cells) and high CRP. Pts had been receiving broad-spectrum antibiotic therapy from the beginning but without effect, and they were changed later according to antibiogram from blood cultures. Antibiotic therapy in all our cases dose not generally cure CRBI so removal of the CVC was recommended, and all CVC were removed. After the insertion of new CVC all clinical signs of infection disappeared and blood culture were sterile.

## Conclusion

In conclusion, the treatment of CRBI caused by *S. maltophilia* must include early and accurate diagnosis, use of effective preventive strategies, and appropriate therapeutic clinical decisions about catheter removal.

## Disclosure of interest

None declared.

